# Supplementary material for: Blood markers of endothelial dysfunction and their correlation to cerebrovascular reactivity in patients with chronic hepatitis C infection
Source: PeerJ. 2021 Jan 14;9:e10723. doi: 10.7717/peerj.10723 (PMC7811780; doi:10.7717/peerj.10723)
Supplement: Supplemental Information 2 [file peerj-09-10723-s002.docx]

Sex: 1 –male, 0- female;

Smoking: 1-yes, 0-no;

Alcohol: 1-yes, 0-no;

Hep C: 1-yes, 0-no;
